# Metastatic spread in patients with gastric cancer

**DOI:** 10.18632/oncotarget.10740

**Published:** 2016-07-20

**Authors:** Matias Riihimäki, Akseli Hemminki, Kristina Sundquist, Jan Sundquist, Kari Hemminki

**Affiliations:** ^1^ Division of Molecular Genetic Epidemiology, German Cancer Research Centre (DKFZ), Heidelberg, Germany; ^2^ Center for Primary Health Care Research, Lund University, Malmö, Sweden; ^3^ Cancer Gene Therapy Group, Faculty of Medicine, University of Helsinki, Finland; ^4^ Helsinki University Hospital Comprehensive Cancer Center, Helsinki, Finland

**Keywords:** gastric cancer, metastasis, epidemiology

## Abstract

**Background:**

The epidemiology of metastatic gastric cancer is unexplored because cancer registries seldom cover metastatic involvement apart from “present or not”. We used a novel approach by utilizing Swedish registers to assess metastatic spread in gastric cancer. To our knowledge, this is the first nationwide description of metastases in gastric cancer.

**Results:**

The most common sites of metastasis were liver (in 48% of metastatic cancer patients), peritoneum (32%), lung (15%), and bone (12%). Metastases to the lung, nervous system, and bone were more frequent in cardia cancer and men, whereas non-cardia cancer more frequently metastasized within the peritoneum. Signet ring adenocarcinomas more frequently metastasized within the peritoneum, bone and ovaries, and less frequently to the lungs and liver compared with generic adenocarcinoma. The liver and the peritoneum were commonly single metastases while lung metastases occurred frequently together with liver metastases. The median survival in metastatic gastric cancer was 3 months, worst among those with bone and liver metastases (2 months).

**Methods:**

A total of 7,559 patients with gastric cancer were identified. Metastatic patterns and survival depending on sex, age, stage, anatomical location (cardia and non-cardia), and histological type were assessed.

**Conclusions:**

The patterns of metastasis differ notably depending on histological type. Cardia cancer exhibits a completely different metastatic behavior than non-cardia cancer. Awareness of the differing patterns may guide in tailored diagnosis of metastases. Survivors from cardia cancer would benefit from increased surveillance of extraperitoneal metastases. Bone metastases should be considered in patients with signet ring adenocarcinoma if symptoms emerge.

## INTRODUCTION

Gastric cancer is the fourth most common cancer worldwide and it ranks second among cancer deaths [1]. Metastatic spread is fatal, causing mass-effect and failing of physiological homeostasis. During the last two decades, the proportion of gastric cancer patients that present with metastases has increased to over 40 % [2–4]. Although the age-adjusted incidence and mortality rates in gastric cancer have decreased during the last decades, the relative survival has only witnessed a modest increase, compared to improvements in many other gastrointestinal cancers [5]. In selected patients receiving more aggressive treatment in selected hospital based trials, even metastatic gastric cancer can confer a median survival of 16 months, [6] contrasting the 3–4 months in most population based studies [2, 3].

Cancer registries seldom include information on metastases apart from the M stage at diagnosis (i.e. “present” or “not present”), thus not allowing investigation of site-specific metastases. As a result, population based epidemiology of metastases from gastric cancer is virtually unknown. This is contrasted by the recent progress in metastasis at the cellular and molecular levels [7, 8]. Overviews of metastatic patterns across different cancers are limited to autopsy-based studies relying on approximately one thousand deaths from metastatic cancer [9–11]. Specific autopsy based reports from the 1970's and 1980's also exist for gastro-intestinal cancers, helping to map metastatic pathways [12, 13]. However, the clinical importance of autopsy reports may not be self-evident, because cancer may spread rapidly in the palliative phase which seldom can be treated or prevented [14].

As alternative approaches to investigate the patterns of metastasis we used here information from Swedish nationwide medical registers. We describe patterns of metastasis from gastric cancer to specific sites, depending on sex, stage, age at diagnosis, histological subtype, and the anatomical location of the primary cancer in the stomach. We also assess survival in metastatic gastric cancer. To our knowledge, this is the first time metastatic gastric cancer is investigated on a nationwide level and its metastatic patterns characterized.

## RESULTS

We identified all patients with gastric cancer diagnosed between 2002 and 2012 (*N* = 8,321). Patients with GIST (*N* = 428), carcinoids (169), unknown histology (*N* = 68), and patients with other histological types (97) were omitted from this study. Thus, 7,559 patients remained for analysis. The primary site was in cardia (1,939), fundus or corpus (1,784), pylorus or antrum (1,480), or unknown (2,356). Of all patients with gastric cancer, 1,945 (26%) had metastasis to a single site, and 980 (13%) had metastasis to multiple sites. The most common sites of metastasis were the liver (in 48%), peritoneum (32%), and lung (15%). A further 857 patients (11%) had metastases to lymph nodes, ill-defined, or unspecified sites, bringing the total to 50%. The number of patients, and their sex, histological subtype, and stage are summarized in Table 1. Patients with cardia cancer were younger compared with patients with non-cardia cancer. Women and patients with other than cardia cancer more often had signet ring adenocarcinoma.

**Table 1:** Overview table of patient characteristics and median age at diagnosis (years) in patients with gastric cancer diagnosed between 2002 and 2012

	Cardia				Corpus/Fundus				Antrum/Pylorus				Unknown				Total	
	Men		Women		Men		Women		Men		Women		Men		Women			
	*N*	Col %	*N*	Col %	*N*	Col %	*N*	Col %	*N*	Col %	*N*	Col %	*N*	Col %	*N*	Col %	*N*	Col %
Total (Row %)	1488	20%	451	6%	1006	14%	778	11%	794	11%	686	9%	1351	18%	1005	14%	7559	100%
Median age in years	68		72		74		75		75		76		74		76		73	
Stage at diagnosis*																		
*N0M0*	263	25%	76	27%	218	31%	183	35%	205	37%	192	41%	189	27%	139	27%	1465	31%
*N + M0*	385	37%	90	32%	182	26%	141	27%	190	34%	146	31%	171	25%	117	23%	1422	30%
*M1*	398	38%	118	42%	296	43%	199	38%	159	29%	134	28%	328	48%	252	50%	1884	39%
Histology																		
*Adenocarcinoma*	1372	92%	411	91%	881	88%	627	81%	692	87%	573	84%	1169	87%	800	80%	6525	86%
*Signet ring*	68	5%	29	6%	99	10%	127	16%	77	10%	85	12%	146	11%	174	17%	805	11%
*Mucinous*	48	3%	11	2%	26	3%	24	3%	25	3%	28	4%	36	3%	31	3%	229	3%

* Stage was missing for 2788 patients.

**Figure 1:**
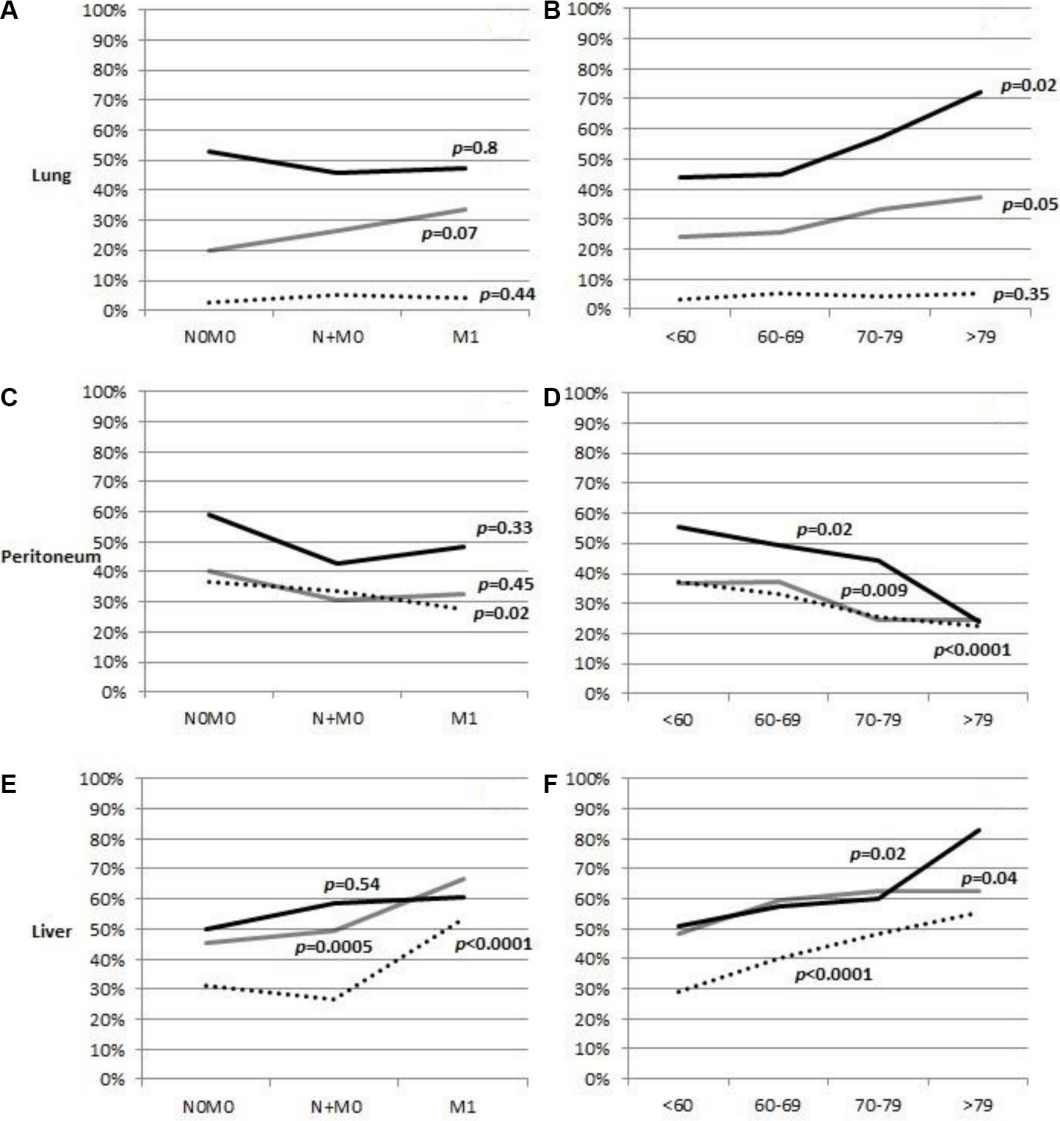
Frequency of lung, peritoneal, and liver metastases in patients with gastric cancer, depending on how many metastases were present Panels (**A**, **C**, and **E**) display trend over stages, and panels (**B**, **D**, and **F**) display trend over the age at diagnosis of patients. Dotted line: one metastasis, gray line: two metastases, black line: three or more metastases. 100% = All patients with metastases. *P*-value for difference between stage or age.

The relative frequency of specific metastases depending on stage and age at diagnosis is addressed in Supplementary Tables 1 and 2. Separate comparisons are available depending on the number of metastases. We chose to highlight the distribution of these variables for lung, peritoneal, and liver metastases in Figure 1. There was very little variation in the relative frequency lung or peritoneal metastases depending on stage (Figure 1A and 1C). However, liver metastases were relatively more common as solitary metastases in patients with stage M1 than in patients without distant metastasis at diagnosis (Figure 1E). Liver metastases were also more common in older patients, irrespective of the number of metastases (Figure 1F). Across age groups, lung metastases followed a similar pattern, being more common in older patients (Figure 1B), whereas peritoneal metastases were more common in younger patients (Figure 1D).

Table 2 shows ORs of metastasis to a single organ. Compared with cardia cancer, non-cardia cancer more frequently metastasized within the peritoneum (OR = 1.7 [fundus and corpus]/1.8 [antrum and pylorus]), as did mucinous and signet ring adenocarcinomas (2.1). Compared with generic adenocarcinoma, signet ring adenocarcinoma metastasized less to the thorax (0.4) and liver (0.3), but more to the bones (2.1). The OR for liver metastases was 0.8 for women versus men.

**Table 2:** Multivariable logistic regression model for ORs of specific metastases in gastric cancer patients with a single metastasis (*N* = 1,945)

Patient characteristics	Any metastasis			Thorax				Peritoneum				Liver				Other Gastro-intestinal				Bone				Other			
	OR	95 % CI		%	OR	95 % CI		%	OR	95 % CI		%	OR	95 % CI		%	OR	95 % CI		%	OR	95 % CI		%	OR	95 % CI	
All				5%				29%				44%				5%				7%				10%			
Sex																											
*Men*	1			5%	1			26%	1			48%	1			4%	1			8%	1			8%	1		
*Women*	1.0	0.9	1.1	3%	0.6	0.4	0.9	34%	1.1	1.0	1.4	37%	0.8	0.7	0.9	7%	1.5	1.0	2.2	6%	0.7	0.5	1.0	13%	1.8	1.3	2.4
Age at diagnosis																											
*< 60*	1			3%	1			37%	1			29%	1			6%	1			12%	1			14%	1		
*60 – 69*	0.9	0.8	1.1	5%	1.7	0.9	3.4	33%	0.9	0.7	1.1	40%	1.3	1.0	1.6	5%	0.8	0.5	1.5	8%	0.7	0.4	1.0	8%	0.6	0.4	0.9
*70 – 79*	0.7	0.6	0.9	4%	1.4	0.7	2.7	26%	0.5	0.4	0.7	48%	1.4	1.1	1.7	6%	0.8	0.5	1.4	5%	0.4	0.2	0.6	10%	0.7	0.5	0.9
*> 79*	0.4	0.3	0.5	6%	1.1	0.6	2.3	23%	0.3	0.2	0.4	55%	1.0	0.8	1.2	5%	0.5	0.2	0.8	4%	0.2	0.1	0.3	7%	0.3	0.2	0.4
Anatomical site																											
*Cardia*	1			7%	1			18%	1			51%	1			3%	1			8%	1			11%	1		
*Fundus/Corpus*	0.9	0.8	1.1	3%	0.4	0.2	0.7	33%	1.7	1.3	2.2	46%	0.9	0.7	1.1	4%	1.1	0.5	2.1	5%	0.6	0.4	1.1	8%	0.6	0.4	0.9
*Antrum/Pylorus*	0.9	0.8	1.1	4%	0.5	0.3	0.9	34%	1.8	1.3	2.3	40%	0.7	0.6	0.9	7%	1.7	0.9	3.2	9%	0.1	0.7	1.9	7%	0.5	0.3	0.8
*Unknown*	1.2	1.0	1.4	4%	0.7	0.4	1.1	33%	2.0	1.6	2.7	38%	0.9	0.7	1.1	6%	0.9	1.1	3.3	6%	0.9	0.6	1.5	12%	1.1	0.8	1.6
Histology																											
*Adenocarcinoma*	1			5%	1			26%	1			48%	1			5%	1			6%	1			9%	1		
*Signet ring*	1.2	1.0	1.4	2%	0.4	0.2	1.1	56%	2.5	2.0	3.1	11%	0.3	0.2	0.4	7%	1.4	0.8	2.5	12%	2.1	1.3	3.2	12%	1.3	0.9	2.0
*Mucinous*	0.7	0.5	1.0	2%	0.3	0.0	2.3	27%	0.8	0.5	1.5	45%	0.7	0.4	1.1	16%	2.7	1.2	5.8	0%				9%	0.8	0.3	2.1

The model adjusts for sex, age at diagnosis, anatomical site of primary, and histological type

Bold values indicate significantly higher ORs, and underlined values indicate significantly lower ORs.

ORs of specific metastases, irrespective of the number of metastases, are displayed in Table 3. Compared with cardia, non-cardia primaries metastasized less to the lung (0.5/ 0.5), liver (0.8/ 0.6), bone (0.7/ 0.4), and nervous system (0.5/ 0.1), but more frequently within the peritoneum (1.8/ 1.6). Lung metastases were more common than peritoneal metastases in cardia cancer (23% vs 20 %), whereas peritoneal metastases were threefold as common as lung metastases in non-cardia cancer. As in Table 2, histological type affected metastasis. Compared with generic adenocarcinoma, signet ring adenocarcinoma more frequently metastasized within the peritoneum (2.3), and less frequently to the lungs (0.4) and liver (0.3). Mucinous adenocarcinoma metastasized more to the pleura and mediastinum compared with generic adenocarcinoma (2.2). Men had more liver metastases and women had more peritoneal metastases.

**Table 3:** Multivariable logistic regression model for ORs of specific metastases in gastric cancer patients with any number of metastases

Patient characteristics	Any metastasis			Lung				Pleura/Mediastinum				Peritoneum				Liver				Other Gastro-intestinal				Nervous system				Bone				Other				% with metastases
	OR	95 % CI		%	OR	95 % CI		%	OR	95 % CI		%	OR	95 % CI		%	OR	95 % CI		%	OR	95 % CI		%	OR	95 % CI		%	OR	95 % CI		%	OR	95 % CI		
All				15%				6%				32%				48%				10%				3%				12%				17%				39%
Sex																																				
*Men*	1			17%	1			5%	1			28%	1			54%	1			9%	1			4%	1			12%	1			14%	1			39%
*Women*	1.0	0.9	1.1	12%	0.8	0.6	1.0	6%	1.2	0.9	1.7	38%	**1.3**	1.1	1.5	39%	0.7	0.7	0.8	11%	1.2	1.0	1.6	3%	1.1	0.7	1.7	11%	1.0	0.8	1.2	23%	**1.6**	1.3	2.0	37%
Age at diagnosis																																				
*<60*	1			15%	1			8%	1			40%	1			38%	1			12%	1			5%	1			19%	1			23%	1			51%
*60-69*	0.9	0.7	1.0	15%	0.9	0.7	1.2	6%	0.7	0.4	1.0	36%	0.8	0.7	1.0	47%	1.1	1.0	1.4	8%	0.6	0.5	0.9	5%	0.9	0.6	1.5	14%	0.7	0.5	0.9	17%	0.7	0.5	0.9	47%
*70-79*	0.6	0.5	0.7	15%	0.8	0.6	1.0	5%	0.5	0.3	0.7	27%	0.5	0.4	0.6	52%	1.1	0.9	1.3	9%	0.5	0.4	0.7	2%	0.4	0.2	0.7	9%	0.4	0.3	0.5	17%	0.5	0.4	0.7	39%
*>79*	0.3	0.3	0.4	15%	0.5	0.4	0.7	3%	0.2	0.1	0.3	23%	0.2	0.2	0.3	58%	0.7	0.6	0.8	10%	0.4	0.3	0.5	1%	0.1	0.0	0.3	5%	0.1	0.1	0.2	12%	0.2	0.2	0.3	24%
Anatomical site																																				
*Cardia*	1			23%	1			7%	1			20%	1			59%	1			7%	1			7%	1			15%	1			16%	1			42%
*Fundus/Corpus*	0.9	0.8	1.0	12%	0.5	0.4	0.6	6%	0.8	0.5	1.2	38%	**1.8**	1.4	2.2	50%	0.8	0.7	0.9	10%	1.2	0.9	1.8	3%	0.5	0.3	0.8	10%	0.6	0.5	0.9	19%	1.1	0.8	1.4	36%
*Antrum/Pylorus*	0.8	0.7	0.9	13%	0.5	0.4	0.7	3%	0.4	0.2	0.7	36%	**1.6**	1.2	2.0	44%	0.6	0.5	0.7	12%	1.4	1.0	2.0	1%	0.1	0.1	0.4	11%	0.7	0.5	1.0	16%	0.9	0.6	1.1	34%
*Unknown*	1.0	0.9	1.2	12%	0.6	0.4	0.7	6%	0.8	0.5	1.2	36%	**1.8**	1.5	2.2	41%	0.7	0.6	0.8	10%	**1.4**	1.0	2.0	2%	0.3	0.2	0.6	11%	0.8	0.6	1.0	19%	1.2	0.9	1.5	40%
Histology																																				
*Adenocarcinoma*	1			16%	1			5%	1			28%	1			53%	1			9%	1			4%	1			11%	1			17%	1			38%
*Signet ring*	1.1	1.0	1.3	6%	0.4	0.3	0.7	9%	**1.9**	1.2	2.8	58%	**2.3**	1.9	2.7	16%	0.3	0.2	0.4	11%	1.2	0.8	1.7	3%	0.9	0.5	1.9	17%	**1.6**	1.2	2.2	21%	1.2	0.9	1.6	43%
*Mucinous*	0.8	0.6	1.1	22%	1.2	0.7	2.0	11%	**2.2**	1.1	4.3	33%	1.0	0.7	1.6	43%	0.7	0.5	1.0	16%	1.6	0.9	2.9	3%	0.6	0.2	2.6	15%	1.2	0.7	2.2	22%	1.2	0.7	1.9	35%

The model adjusts for sex, age at diagnosis, anatomical site of the primary, and histological type

Bold values indicate significantly higher ORs, and underlined values indicate significantly lower ORs.

Table 4 addresses the distribution of metastases, separately for cardia cancer, non-cardia cancer, and all patients with gastric cancer. More than half of patients with lung metastases also had liver metastases. Women with ovarian metastases frequently had peritoneal metastases (in 56% of all). Gastric cancer patients with pleural/mediastinal metastases also often had peritoneal metastases (32%). Patients with nervous system metastases often also had lung metastases (in 21% of all), but seldom had peritoneal metastases (in 9%). Some differences can be seed between cardia and non-cardia cancer. For example, 42% of patients with pleural/mediastinal also had peritoneal metastases in non-cardia cancer, but only 16% on cardia cancer.

**Table 4:** Location of second metastasis in gastric cancer patients with one or multiple metastases (two or more)

Site of metastasis	Total amount	As one of N listed metastases				% multiple	Lung	Pleura/Mediastinum	Peritoneum	Liver	Other G-I	Nervous system	Bone	Ovary*	Other
		1	2	3	4+										
**ALL**															
*Lung*	440	89	191	111	49	80%	100%	9%	16%	55%	8%	5%	13%	1%	16%
*Pleura/Med*.	161	9	50	50	52	94%	25%	100%	32%	25%	3%	3%	16%	6%	18%
*Peritoneum*	934	570	209	104	51	39%	8%	6%	100%	16%	7%	1%	4%	5%	12%
*Liver*	1416	848	381	136	51	40%	17%	3%	11%	100%	6%	2%	7%	1%	10%
*Other G-I*	279	102	112	50	15	63%	12%	2%	23%	30%	100%	2%	7%	2%	13%
*Nervous system*	98	37	32	15	14	62%	21%	5%	9%	27%	6%	100%	18%	1%	16%
*Bone*	350	136	109	64	41	61%	16%	7%	11%	29%	6%	5%	100%	4%	21%
*Ovary*	82	20	29	18	15	76%	7%	12%	56%	12%	7%	1%	18%	100%	21%
*Other*	445	134	163	93	55	70%	16%	7%	26%	31%	8%	4%	17%	4%	100%
**CARDIA**															
*Lung*	188	35	85	46	22	81%	100%	8%	13%	60%	7%	6%	14%	0%	15%
*Pleura/Med*.	55	5	17	19	14	91%	27%	100%	16%	40%	2%	5%	13%	0%	15%
*Peritoneum*	168	90	42	25	11	46%	14%	5%	100%	26%	4%	1%	6%	1%	12%
*Liver*	481	253	151	56	21	47%	23%	5%	9%	100%	5%	4%	10%	0%	10%
*Other G-I*	59	17	25	11	6	71%	24%	2%	12%	37%	100%	7%	10%	0%	15%
*Nervous system*	54	19	21	6	8	65%	22%	6%	4%	33%	7%	100%	13%	2%	17%
*Bone*	123	40	42	25	16	67%	22%	6%	8%	38%	5%	6%	100%	1%	24%
*Ovary*	4	1	0	3	0	75%	0%	0%	50%	25%	0%	25%	25%	100%	25%
*Other*	125	34	39	30	22	73%	23%	6%	16%	40%	7%	7%	24%	1%	100%
**NONCARDIA**															
*Lung*	139	25	60	39	15	82%	100%	7%	21%	56%	9%	3%	11%	2%	21%
*Pleura/Med*.	53	2	17	18	16	96%	19%	100%	42%	21%	2%	0%	15%	9%	15%
*Peritoneum*	428	259	100	46	23	39%	7%	5%	100%	17%	7%	1%	4%	7%	12%
*Liver*	544	336	137	50	21	38%	14%	2%	13%	100%	8%	1%	5%	1%	10%
*Other G-I*	123	43	47	26	7	65%	11%	1%	24%	35%	100%	1%	9%	2%	15%
*Nervous system*	25	9	7	6	3	64%	16%	0%	12%	20%	4%	100%	36%	0%	20%
*Bone*	122	53	32	23	14	57%	12%	7%	15%	23%	9%	7%	100%	5%	21%
*Ovary*	41	9	16	7	9	78%	7%	12%	68%	15%	7%	0%	15%	100%	15%
*Other*	170	39	72	42	17	77%	17%	5%	31%	33%	11%	3%	15%	4%	100%

Percentages indicate how many of all patients with site-specific metastases have multiple metastases to other sites.

**Figure 2:**
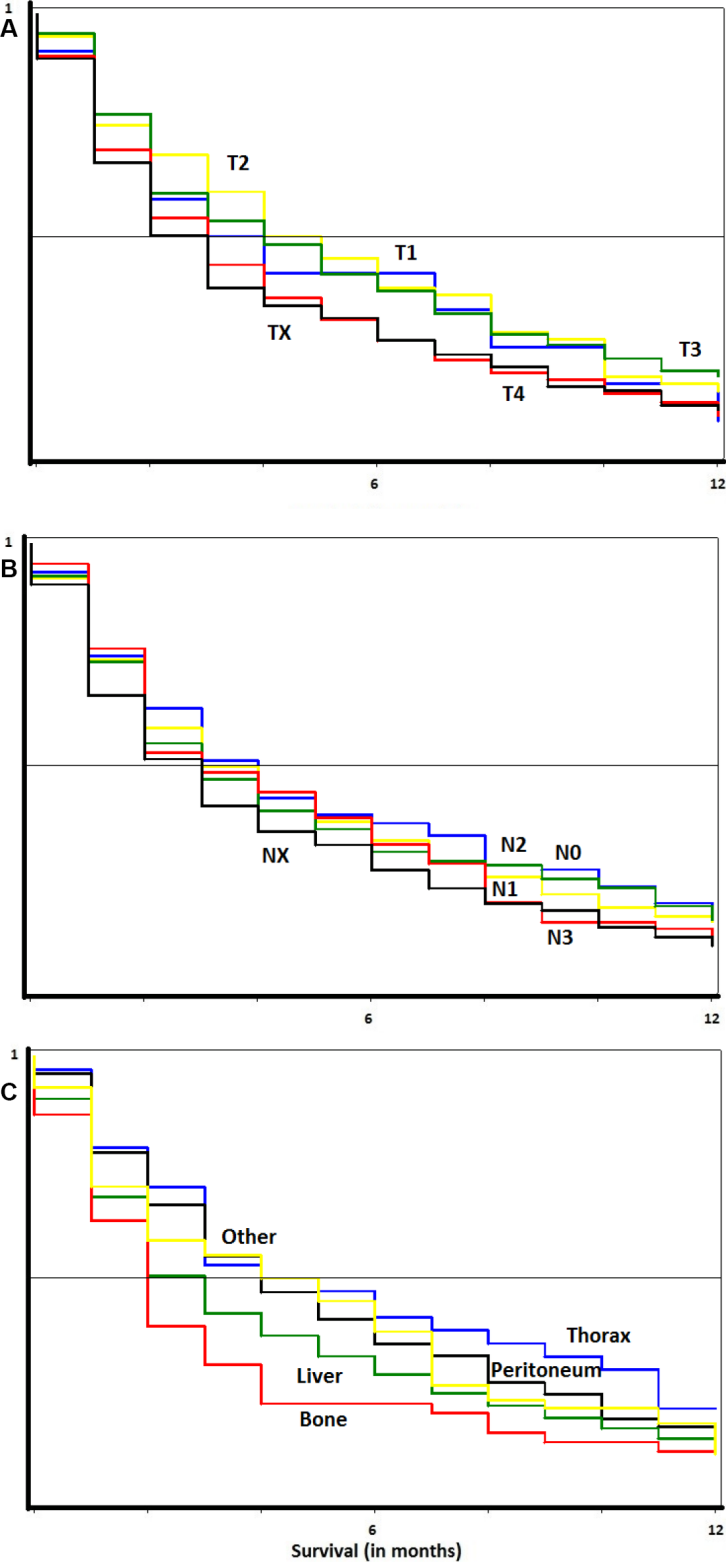
Survival curves in metastatic gastric cancer In panel (**A**) comparison by T stage. In panel (**B**) comparison by N stage. In panel (**C**) comparison by site of metastasis.

Survival after diagnosis of metastatic gastric cancer is depicted in Table 5. All patients in this analysis were metastatic at diagnosis (i.e., staged M1) and had metastasis to one site only. Patients diagnosed at age younger than 60 had a median survival at 6 months. Compared to them, older patients fared worse, with median survivals of 3 months (HR = 1.28) if aged 60–69, 3 months (1.58) if aged 70–79, and 2 months (2.28) if aged 80 or older at diagnosis. No substantial difference in survival could be seen depending on N stage, sex, or metastatic site. Low T stage was a good prognostic factor. Survival curves after diagnosis of metastatic gastric cancer with solitary metastases are depicted in Figure 2, depending on A) T stage, B) N stage, and C) site of metastasis. The survival curve for stage TX closely follows that for stage T4, whereas survival for stages T1–T3 was somewhat better. Survival did not differ notably between N stages, although it was worst in stage NX. More notable differences could be seen depending on site of metastasis. Survival was clearly worst for those with liver or bone metastases, whereas it was better in thoracic metastases.

**Table 5:** Median survival (months)and multivariable HR for death after diagnosis of metastatic (M1) gastric cancer, by sex, primary site, histological subtype, stage, age at diagnosis, and metastatic site

	Patients with one metastasis				
Characteristic	*N*	Median survival (months)	HR	95 % CI	
**Total**	818	3			
**Sex**					
*Men*	535	3	1	1	1
*Women*	283	3	1.10	0.94	1.28
**Age at diagnosis**					
*<60*	166	6	1	1	1
*60-69*	221	3	**1.27**	1.03	1.56
*70-79*	259	3	**1.56**	1.27	1.93
*>79*	172	2	**2.26**	1.79	2.86
**T stage**					
*T1*	12	3.5	0.84	0.47	1.50
*T2*	60	4.5	0.64	0.48	0.87
*T3*	265	4	0.77	0.64	0.92
*T4*	256	3	1	1	1
*TX*	225	2	0.90	0.74	1.09
**N stage**					
*N0*	113	3	0.97	0.77	1.23
*N1*	267	3	1	1	1
*N2*	102	3	1.02	0.81	1.29
*N3*	69	3	1.05	0.8	1.38
*NX*	267	3	1.13	0.94	1.35
**Metastatic site**					
*Thorax*	34	4.5	0.73	0.51	1.05
*Other abdominal*	241	4	0.84	0.70	1.01
*Liver*	438	2	1	1	1
*Bone*	46	2	1.32	0.97	1.81
*Other*	59	4	0.80	0.60	1.07
**Anatomical site**					
*Cardia*	207	3	1	1	1
*Fundus, Corpus*	211	3	0.84	0.68	1.03
*Antrum, Pylorus*	140	3	0.85	0.68	1.07
*Unknown*	260	3	1.03	0.85	1.26
**Histological subtype**					
*Adenocarcinoma*	717	3	1	1	1
*Signet ring*	82	3	1.04	0.82	1.34
*Mucinous*	19	2	1.42	0.89	2.27

Only patients with one distant metastasis are included

## DISCUSSION

Several histological classification systems for gastric cancer are used [15]. The Laurén classification divides gastric cancer into intestinal (generic), diffuse (signet ring), and intermediate type, whereas the more recent WHO further separates intestinal type into tubular, papillary, and mucinous adenocarcinoma. Both mucinous and signet ring adenocarcinomas produce mucin, which is extracellular in mucinous adenocarcinoma and intracellular in signet ring adenocarcinoma [15]. Diffuse adenocarcinoma is more poorly differentiated, not clearly associated with gastric metaplasia, and show some genetic similarities to neuroendocrine cancer [16]. The genetics of diffuse adenocarcinoma are not fully understood, but molecular profiling have suggested a different gene expression pattern from intestinal adenocarcinoma.[17]. Peritoneal metastases were twice as common in signet ring adenocarcinoma compared with generic type adenocarcinoma, whereas metastases apparently arisen by hematologic spread were less frequent. Mucus produced by adenocarcinomas may infiltrate the surrounding stroma and help the tumor invading the stroma more rapidly, thus facilitating spread to the serosa and within the peritoneal fluid [18]. There might also be a genetic link between peritoneal growth and mucin production.

Cardia and non-cardia cancers differ significantly from each other with regard to symptoms, geography, risk factors, and pathological features [19, 20]. The incidence of non-cardia cancer has showed a clear decreasing trend, related to the decreasing prevalence of Helicobacter pylori infections, whereas reports on incidence trends in cardia cancer are conflicting. For the definition of the “cardia”, many classification systems have been proposed [21]. Many cardiac gastric resemble esophageal cancer with regard to histology, and treatment is also similar. However, the pattern of lymphatic spread favors grouping of cardia cancer together with non-cardia cancer and not with esophageal cancer which is also acknowledged in current staging systems [21, 22]. Nevertheless, cardia cancer could also be considered a separate entity, [20] supported by the present results showing a substantial difference in the metastatic behavior of cardia and non-cardia cancer. In cardia cancer, lung metastases were twice as common as in non-cardia cancer while the opposite proportion prevailed for peritoneal metastases.

Two hypotheses are widely accepted to explain metastatic spread in cancer [12, 23, 24]. Simplified, the “anatomical/mechanical” hypothesis states that anatomical factors promote specific spreading and the “seed-and-soil” hypothesis implies organ specific tropism of circulating tumor cells. The differing biology between generci and signet ring adenocarcinomas may explain the differing tropisms of metastasis, conforming to the seed and soil hypothesis. Autopsy studies have put forward the concept of cascadic spread of gastro-intestinal tumors, suggesting that metastases at the first draining site may act as seeds to further metastasis [12, 24]. Blood is mainly drained through the portal system via the liver, to the systemic circulation. It is logical that cardia cancer indeed metastasized more frequently to the lungs compared with non-cardia cancer, because blood from the proximal ventricle may flow directly to the lungs, surpassing the liver [12]. According to the present results, cardia cancer was actually more prone to metastasize to the liver, compared with non-cardia cancer, indicating a difference in biology, conforming to the “seed and soil” hypothesis. Most patients with lung metastases also had liver metastases. Patients with pleural or ovarian metastases often had peritoneal metastases. In contrast, patients with liver metastases seldom had peritoneal metastases, indicating that gastric cancer typically metastasizes either within the peritoneum or hematogenically, and seldom by both routes.

There was no difference in survival between the anatomic subtypes, all showing a median survival of only three months. Survival irrespective of N stage, histological type, or sex was three months. Location of metastasis affected survival in that it was only two months in single liver or bone metastases, but 4.5 months in single thoracic metastases. Depending on the age, survival ranged between two and six months. Reports from the Netherlands have described similar overall survival rates [2]. Sadly, survival has not increased over the last decades, [2, 5] calling for improved early detection and treatment. The most recent years have sprung some hope, although no clear consensus exists on the optimal treatment regimens in metastatic gastric cancer [25]. The introduction of HER-2 antibodies has been beneficial, and one may theorize about the possibilities of checkpoint inhibiting antibodies [26].

The liver as an eventual target organ for metastases was relatively less frequent if the cancer was metastatic at diagnosis compared with stage M0. This could be explained by immunological factors and tumor-stroma interactions which may keep liver metastases in a dormant state [7, 8]. In the meantime, metastases may develop at other sites instead. Dormant metastases may be undetectable, and resistant to therapy. The liver features a unique immunological milieu, which may affect the tumor-stroma interactions [27]. Although older patients did not have an increased risk of liver metastases, liver metastases were relatively more frequent in older patients. This may also be an expression of the immunology of the liver. Patients with dominant liver metastases could represent a tumor immunologically distinct from those cases with lung and bone metastases, which may be more readily detected by the immune surveillance in younger patients.

Swedish nationwide registers are known for their coverage and reliability. The completeness of registration to the cancer registry is over 90% [28] and the national patient register reached completeness in national coverage already in 1987 [29]. However, we do not have access to clinical parameters such as time of diagnosis of metastases or imaging modalities: a common shortcoming in cancer epidemiology. When analyzing the frequency of metastases it is important to consider the specified time that metastases are measured. Metastases are naturally less common at diagnosis than at death. Autopsy studies report higher frequencies of metastases in cancer patients and also patients “without” (a diagnosis of) cancer. Malignant or *in situ* tumors are expected in a majority of men and women already in their forties, according to autopsy data [14]. We argue however, that our approach should adequately consider metastases that are clinically relevant.

In conclusion, novel Figures on the metastatic spread from gastric cancer are presented, providing a comprehensive overview of a previously unexplored subject. Cardia cancer has a completely different metastatic behavior than non-cardia cancer, metastasizing more to the lungs, bone, and nervous system, but less within the peritoneum. Compared with generic adenocarcinoma, signet ring adenocarcinomas metastasize twice as often within the peritoneum, but also frequently to the bone. Survival in metastatic gastric cancer is poor irrespective of T- or N-stage, site of metastasis, or anatomical location of gastric cancer. Survival is worst in patients aged over 79 (2 months vs 6 months in patients aged < 60) and those with bone or liver metastases (both 2 months) compared with thoracic metastases (4.5 months). To our knowledge, this is the first time site-specific metastases from gastric cancer are assessed at a population level, and that the differing metastatic patterns in cardia and non-cardia gastric cancer are described. Although the prognosis is indeed dire in gastric cancer, extraperitoneal metastases should be regarded in younger patients diagnosed with cardia cancer, so that treatment can be given as early as possible. Bone metastases should be considered in patients with signet ring adenocarcinoma.

## MATERIALS AND METHODS

This study utilized data from nationwide registers in Sweden. Cancer data was obtained from the Swedish Cancer Registry, which includes all cancer diagnosed in Sweden since 1958 [30]. The primary cancers are coded by the International classification of diseases' (ICD) 10th revision in the Cancer Registry. Patients with gastric cancer were identified with code C16. Then anatomical location was identified through the ICD-10 code: cardia (C16.0), fundus and corpus (C16.1/.2/.5/.6), antrum and pylorus (C16.3/.4). Other patients (C16.9) were listed as “location unknown”. TNM (Tumor-lymph Node-distant Metastasis) staging is available for patients diagnosed since 2002. Patients were also subdivided into three groups depending on the stage at diagnosis of cancer: T_any_N0M0, T_any_N + M0, and T_any_N_any_M +. We identified patients with the histological subtypes signet-ring adenocarcinoma and mucinous adenocarcinoma by the SNOMED codes 8490 and 8480, respectively, whereas generic adenocarcinoma are listed under the code 8140. The latter forms the majority of the “intestinal” type, whereas signet ring corresponds to “diffuse” type (discussed later). Other histological types were excluded. Because stage and histological subtypes is available since 2002 and 1993, respectively, the analysis was restricted to patients diagnosed years 2002 through 2012. Patients were followed until the end of 2012.

Two sources were used for identifying metastatic spread. The National Patient Register includes all hospitalizations in Sweden, with nationwide coverage since 1987, and reporting is obligatory from all healthcare centers, public and private alike [29]. Metastases are listed among one of up to 22 diagnoses during the hospitalization. Causes of death were identified from the national Cause of Death Registry, which includes both the underlying cause and up to ten accompanying causes of death [31]. Since 1996, both registers implement coding through ICD-10. The codes used for identifying metastatic sites were as follows: lung (C78.0), pleura (C78.2), other respiratory organs (C78.1/.3), peritoneum (C78.6), liver (C78.7), other gastro-intestinal (C78.4/.5/.8), urinary system (C79.0/.1), skin (C79.2), nervous system (C79.3/.4), bone (C79.5), ovary (C79.6), adrenal (C79.7), other specified (C79.8). Due to small numbers, urinary system, skin, ovarian, adrenal, and other specified sites were grouped together. Metastases to lymph nodes (C77), “ill-defined” sites (C76), and unspecified sites (C79.9) were not included in this analysis. Patients with multiple primary cancers were excluded. All calculations were performed, and life tables produced, using SAS software, version 9.3 (PROC LOGISTIC, PROC PHREG, PROC LIFETEST).

## SUPPLEMENTARY MATERIALS TABLES


