# Supplementary material for: Metastatic spread in patients with gastric cancer
Source: Oncotarget. 2016 Jul 20;7(32):52307–16. doi: 10.18632/oncotarget.10740 (PMC5239553; doi:10.18632/oncotarget.10740)
Supplement: Supplementary file 1 [file oncotarget-07-52307-s001.pdf]

Metastatic spread in patients with gastric cancer

Supplementary Materials

Supplementary Table S1: Distribution of metastases in stomach cancer by stage and number of metastases

Stage	Number of metastases	Patients with metastases		Lung			Peritoneum			Liver			Other gastro-intestinal			Bone			Nervous system			Other		
		N	%	*N*	%	*p*	*N*	%	*p*	*N*	%	*p*	*N*	%	*p*	*N*	%	*p*	*N*	%	*p*	*N*	%	*p*
N0M0	0	1215																						
	1	183	100%	5	3%		67	37%		57	31%		13	7%		8	4%		5	3%		31	17%	
	2	55	100%	11	20%		22	40%		25	45%		14	25%		7	13%		3	5%		21	38%	
	3+	34	100%	18	53%		20	59%		17	50%		7	21%		12	35%		3	9%		23	68%	
N+M0	0	930																						
	1	335	100%	17	5%		112	33%		89	27%		26	8%		43	13%		9	3%		43	13%	
	2	121	100%	32	26%		37	31%		60	50%		26	21%		18	15%		5	4%		41	34%	
	3+	63	100%	29	46%		27	43%		37	59%		16	25%		20	32%		7	11%		38	60%	
M1	1	818	100%	34	4%	0.44	225	28%	0.02	438	54%	< 0.0001	16	2%	< 0.0001	46	6%	0.14	10	1%	< 0.0001	59	7%	< 0.0001
	2	284	100%	96	34%	0.07	92	32%	0.45	189	67%	0.0005	32	11%	0.004	45	16%	0.88	15	5%	0.83	92	32%	0.09
	3+	139	100%	66	47%	0.8	67	48%	0.33	84	60%	0.54	28	20%	0.69	48	35%	0.91	13	9%	0.91	78	56%	0.22

P-values are two-sided, comparing the proportion of specific metastases between stages, separately depending on the number of metastases. Metastases were diagnosed any period between the initial cancer diagnosis and death.

Supplementary Table S2: Distribution of metastases in gastric cancer by age and number of metastatic sites between diagnosis and death

Age	Number of metastases	Patients with metastases		Lung			Peritoneum			Liver			Other gastro-intestinal			Nervous system			Bone			Other		
		N	%	*N*	%	*p*	*N*	%	*p*	*N*	%	*p*	*N*	%	*p*	*N*	%	*p*	*N*	%	*p*	*N*	%	*p*
< 60	1	397	100%	12	3%		147	37%		116	29%		22	6%		12	3%		46	12%		42	11%	
	2	167	100%	40	24%		61	37%		81	49%		34	20%		8	5%		38	23%		64	38%	
	3+	112	100%	49	44%		62	55%		57	51%		24	21%		12	11%		43	38%		87	78%	
60–69	1	537	100%	28	5%		177	33%		215	40%		25	5%		15	3%		44	8%		33	6%	
	2	214	100%	55	26%		80	37%		127	59%		28	13%		11	5%		40	19%		72	34%	
	3+	111	100%	50	45%		55	50%		64	58%		18	16%		15	14%		33	30%		81	73%	
70–79	1	612	100%	27	4%		156	25%		296	48%		34	6%		8	1%		32	5%		59	10%	
	2	183	100%	61	33%		45	25%		114	62%		29	16%		10	5%		26	14%		68	37%	
	3+	70	100%	40	57%		31	44%		42	60%		12	17%		2	3%		21	30%		52	74%	
> 79	1	399	100%	22	6%	0.32	90	23%	< 0.0001	221	55%	< 0.0001	21	5%	0.9	2	1%	0.02	14	4%	< 0.0001	29	7%	0.05
	2	94	100%	35	37%	0.05	23	24%	0.009	59	63%	0.04	21	22%	0.13	3	3%	0.85	5	5%	0.002	34	36%	0.8
	3+	29	100%	21	72%	0.02	7	24%	0.02	24	83%	0.02	11	38%	0.06	0	0%	0.03	8	28%	0.44	16	55%	0.11

P-values are two-sided, comparing the proportion of specific metastases between age groups, separately depending on the number of metastases.
